# What about the buccal surfaces? Dental microwear texture analysis of buccal and occlusal surfaces refines paleodietary reconstructions

**DOI:** 10.1002/ajpa.24509

**Published:** 2022-03-08

**Authors:** Raquel Hernando, John C Willman, Antoine Souron, Artur Cebrià, F. Xavier Oms, Juan I. Morales, Marina Lozano

**Affiliations:** ^1^ Departament d'Història i Història de l'Art Universitat Rovira i Virgili Tarragona Spain; ^2^ Institut Català de Paleoecologia Humana i Evolució Social (IPHES‐CERCA), Zona Educacional 4, Campus Sescelades URV (Edifici W3) Tarragona Spain; ^3^ Laboratory of Prehistory, CIAS – Research Centre for Anthropology and Health, Department of Life Sciences University of Coimbra Coimbra Portugal; ^4^ UMR CNRS 5199, PACEA ‐ Laboratoire de la Préhistoire à l'Actuel: Culture, Environnement et Anthropologie University of Bordeaux Bordeaux France; ^5^ SERP, Departament d'Historia i Arqueologia Universitat de Barcelona Barcelona Spain

**Keywords:** buccal, Cova de la Guineu, DMTA, late Neolithic‐chalcolithic, occlusal

## Abstract

**Objectives:**

This study analyzes and compares dental microwear textures on occlusal and buccal surfaces from the same tooth to determine if using these surfaces in tandem can provide complementary data for dietary reconstructions.

**Materials and methods:**

Cova de la Guineu is a Late Neolithic‐Chalcolithic burial cave located in Font‐Rubí (Barcelona, Spain). The study sample consisted of 69 individuals represented by the lower left second molar. However, only 27 individuals had well‐preserved surfaces. Dental Microwear Texture Analysis was performed on both surfaces using a Sensofar® S Neox white‐light confocal profilometer following standard procedures. Toothfrax® software was used to quantify surface complexity and anisotropy.

**Results:**

The bootstrap resampling analysis shows significant differences in complexity and anisotropy between surfaces. There is no correlation between surfaces for complexity or anisotropy. The occlusal surfaces exhibit high complexities and low anisotropies, which are similar to values observed in Late Neolithic farming groups from Belgium.

**Discussion:**

The combination of occlusal and buccal microwear signatures provided important inferences regarding the studied sample. First, occlusal complexity and anisotropy values indicate an abrasive dietary regime. Second, we propose that the higher anisotropy values found on buccal surfaces, compared to those on the occlusal ones, are attributed to the specific mechanisms of microwear formation for each surface. Finally, combining both surfaces may increase the number of samples suitable for analysis. Further studies, with greater intergroup sampling, will help to understand how buccal microwear reflects or complements DMTA signatures on the occlusal surfaces.

## Graphical abstract

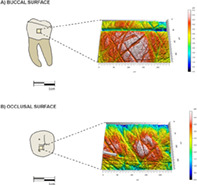

## 1 INTRODUCTION

The quantification of dental microwear—the microscopic wear features found on enamel surfaces—is an effective and non‐destructive method for dietary reconstruction. These wear features are produced, and undergo turnover, during mastication when the compression and movement of the bolus causes abrasion (tooth‐food‐tooth) and attrition (tooth‐to‐tooth). (Maier & Schneck, 1982; Gordon, 1988; Puech, 1979; Teaford & Lytle, 1996, Krueger et al., 2008; Tausch, 2015; Schmidt et al., 2019). Experimental studies have provided greater understanding of the formation and turnover of dental microwear, but many issues remain unresolved. For instance, some authors suggested that the material properties of the food are responsible for dental microwear, and particles softer than the enamel can cause enamel removal (e.g., phytoliths; Rodriguez‐Rojas et al., 2020; Xia et al., 2015). In contrast, nanoscale experimental studies have challenged this argument, suggesting that metallic proxies are not appropriate for understanding microwear formation from a mechanical perspective (van Casteren et al., 2018). Further arguments have posited that food material properties (e.g., toughness and hardness) are less important than their mechanical properties (e.g., shape) and abrasive particle (e.g., phytoliths, dust, grit) content for the formation of microwear (Lucas et al., 2013; van Casteren et al., 2020). However, controlled chewing experiments that replicate dental microwear formation using Artificial Resynthesis Technology (ART‐5) show that grit‐free meat can cause some degree of occlusal microwear, whereas grit‐laden meat generates faster microwear turnover (Krueger et al., 2021). These studies illustrate that dental microwear is a highly complex process with many factors to consider (Teaford et al., 2020). For this reason, further studies are required to better understand this process and how it is expressed on the different types of dental surfaces.

Dental microwear is generally analyzed on either the buccal or occlusal surfaces, and both surfaces are rarely examined in the same study (García‐González et al., 2015; Hernando et al., 2021; Hernando, Willman, et al., 2020). This may be explained by a difference in imaging technology and associated difficulties of data comparison. For instance, occlusal microwear analyses shifted towards quantification through Dental Microwear Texture Analysis (DMTA) using confocal microscopy (e.g., El‐Zaatari, 2010; Mahoney et al., 2016; Schmidt et al., 2015; Schmidt et al., 2019; Ungar et al., 2003), whereas the majority of buccal microwear analyses continue to rely on quantification from images obtained with scanning electron and optical microscopy—SEM and OM, respectively (e.g., Lalueza et al., 1996; Pérez‐Pérez et al., 1994; Romero et al., 2012; Romero et al., 2013; Hernando, Fernández‐Marchena et al., 2020). Recent studies used SEM and OM to demonstrate how the combination of buccal and occlusal microwear can provide complementary data for reconstructing dietary behaviors given the differential rates of microwear accumulation and turnover between the two surfaces (García‐González et al., 2015; Hernando Ackermans et al., 2021; Hernando, Willman et al., 2020). Nonetheless, DMTA has rarely been explored on buccal surfaces (but see: Aliaga‐Martínez et al., 2017), and no study has yet used DMTA on both the occlusal and buccal surfaces of the same tooth.

This study is the first to examine both occlusal and buccal molar microwear textures using the same individual within a single sample. Using occlusal texture analysis, we provide a paleodietary assessment of the Cova de la Guineu sample. We also present a critical examination of DMTA for buccal surfaces, and how their use may refine dietary reconstructions when combined with occlusal microwear studies. Likewise, we suggest that studies integrating data from both surfaces could help understand the interaction of abrasion and attrition contained in microwear signatures.

## 2 MATERIAL

**FIGURE 1:**
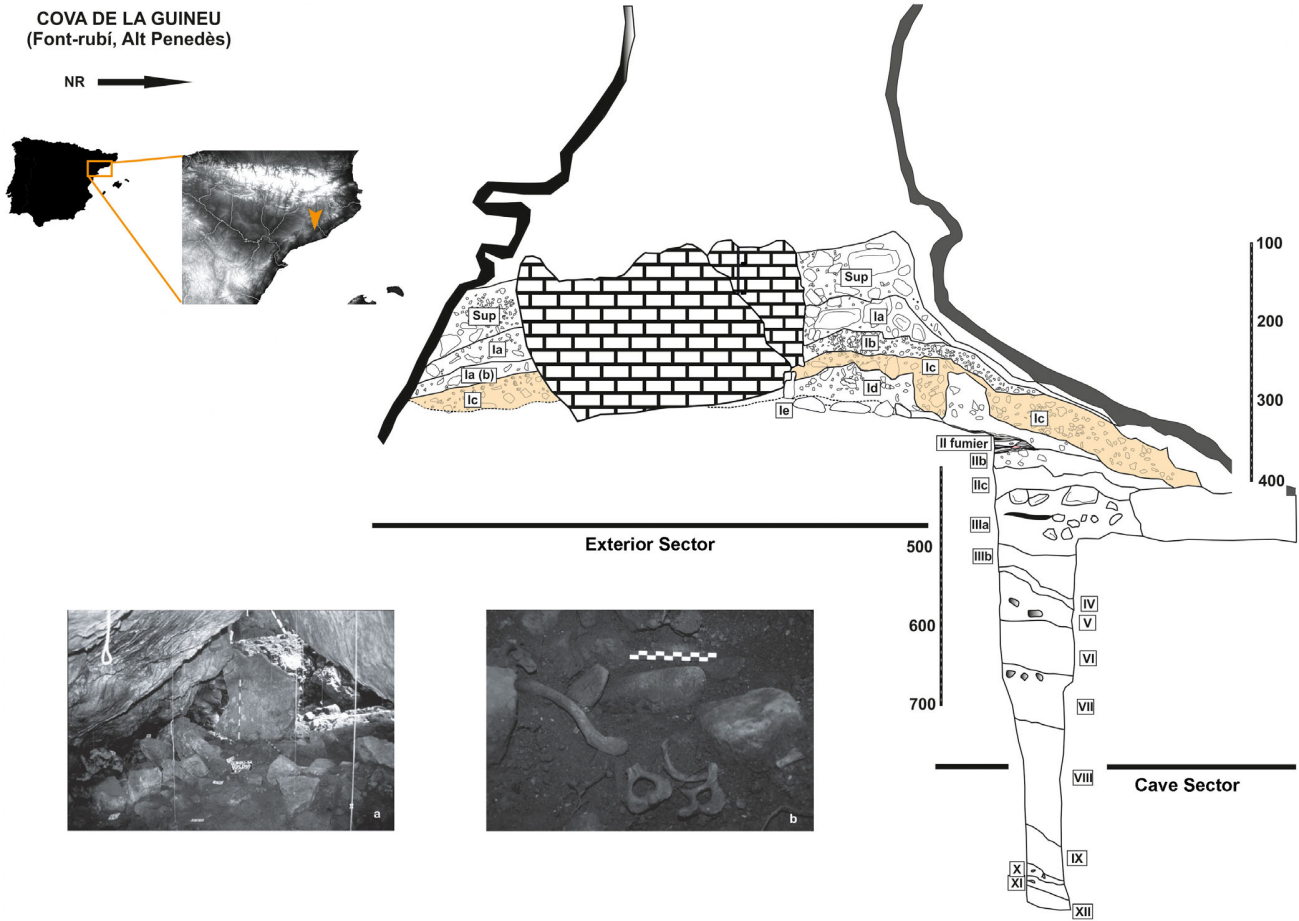
Location of Cova de la Guineu, and the main section. Picture (a) Para‐dolmenic structure; (b) some in situ human remains associated with the funerary phase

The dental remains used in this study are from Cova de la Guineu, a sepulchral cave located in Font‐Rubí, Barcelona, northeastern Iberian Peninsula at 738 m above sea level (m.a.s.l), dated to the Late Neolithic‐Chalcolithic (4820–4454 cal. BP; 5040–4865 cal. BP) (Oms et al., 2016). The funerary phase (level Ic interior) is a collective burial chamber with a para‐dolmenic structure (Figure 1). Most of the human remains were found in disarticulation due to the sequential process of internments in prehistory as well as some historic disturbances of the cave through clandestine activities (Oms et al., 2016).

Left lower second molars (LLM2) are the most abundant adult teeth from Cova de la Guineu, accounting for a minimum number of 69 individuals (Supplementary Table S1). These 69 LLM2s were chosen for buccal and occlusal DMTA. Most of the teeth were isolated but there were also some mandibles and hemimandible fragments among the remains (see Supplementary Figure S1).

## 3 METHODOLOGY

### 3.1 Dental microwear texture analysis

The teeth were cleaned with a soft brush and acetone to remove adherent grit, dust, or adhesive residues (Galbany et al., 2004). Polyvinylsiloxane (Coltène President Plus Jet light body) was used for molding of the occlusal and buccal surfaces of each tooth. A first impression was made to remove any particles that remained on the surface, and the second impression was analyzed (Mahoney et al., 2016). Positive (epoxy) casts were not created since surface detail can be lower in casts compared to direct analysis of the impression material (Mihlbachler et al., 2019). Thus, analyses were conducted directly from silicone impressions (see also: Bas et al., 2020; Mahoney et al., 2016; Ramdarshan et al., 2017). Molds of the buccal and occlusal surfaces were observed and measured with a Sensofar® S Neox microscope driven by the SensoSCAN® software 6.2 at PACEA (University of Bordeaux, France).

**FIGURE 2:**
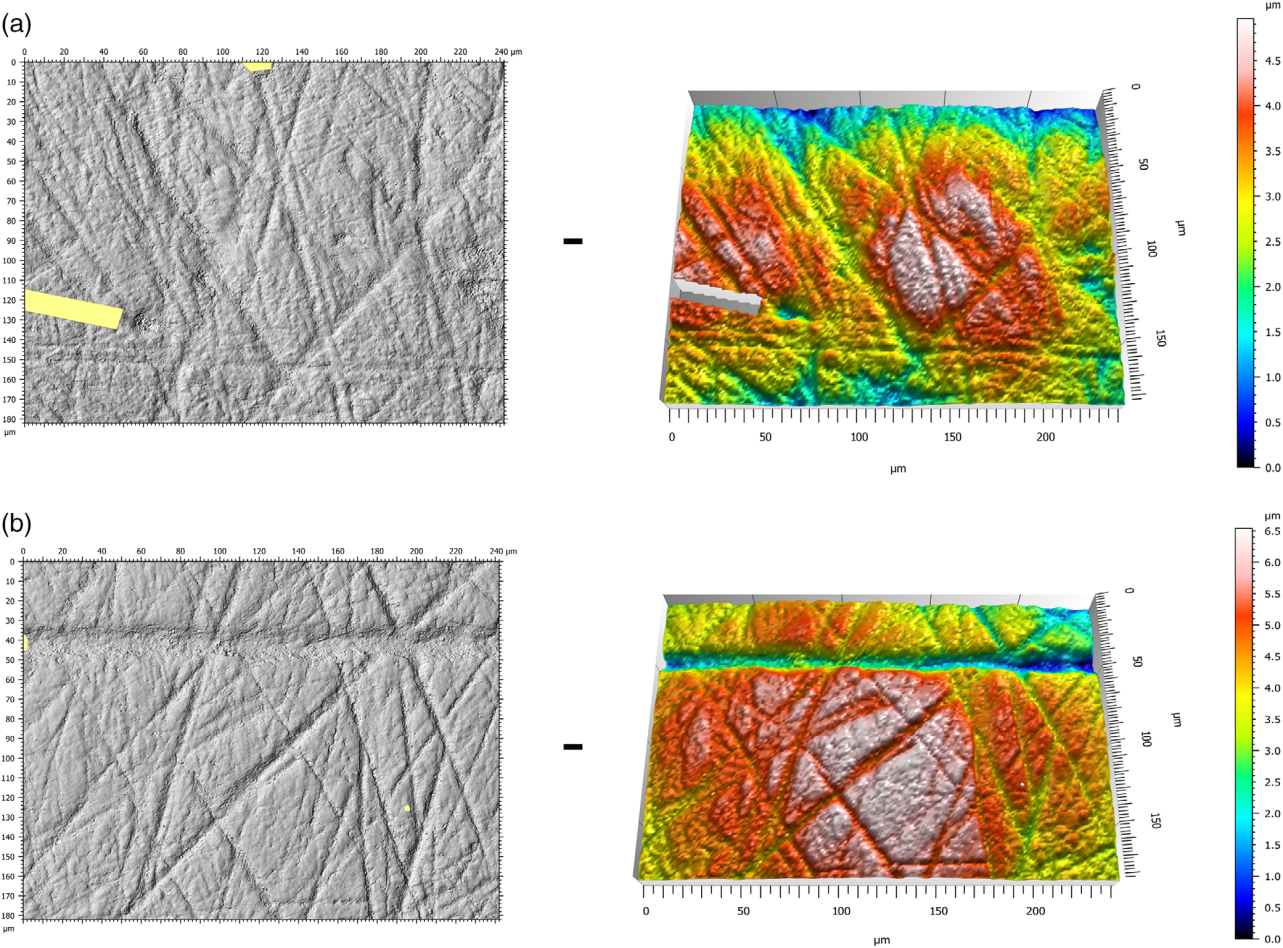
3D views and photosimulations from DMTA on both occlusal (a) and buccal (b) surfaces from an individual of Cova de la Guineu (GN‐1564)

**FIGURE 3:**
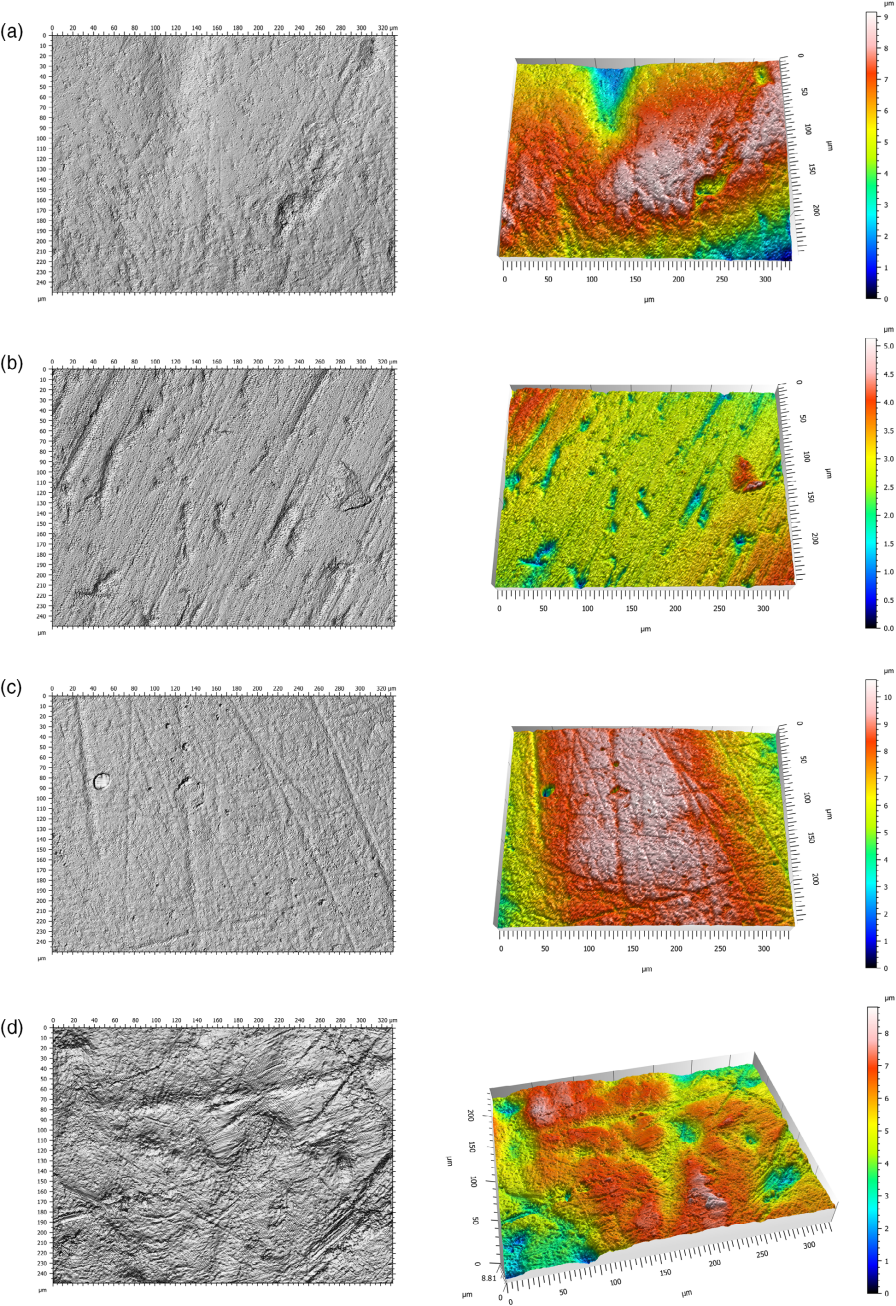
Examples of poor quality DMTA scans that obscured dental microwear features. (a) Occlusal surface (GN91‐REM‐3303): Poor quality mold; (b) occlusal surface (GN95‐REM‐9): Taphonomic features; (c) buccal surface (GN89‐REM‐3611): Mold affected by bubbles; (d) buccal surface (GN89‐REM‐3612): Perikymata

Occlusal surface DMTA was collected on facet 9 for each LLM2 (Scott et al., 2006). SEM analyses of buccal microwear focus on the middle third of the buccal surface (Galbany et al., 2004), so the same area was chosen for buccal DMTA (Aliaga‐Martínez et al., 2017) (Figure 2). The molds of both surfaces were positioned as flat as possible under the microscope in standardized orientation for each tooth and surface. Initial observations were done at 5× magnification to find the area of interest on facet 9 or the buccal middle third (Schmidt et al., 2019). The surface data were measured at 100×, corresponding to an area of 332.3×249.9 μm integrated in four fields of view. The study area was cropped to 242×182 μm to allow broader comparisons with published data for the occlusal surface (e.g., Schmidt et al., 2015, 2019; Williams et al., 2020) and maintain equivalence between occlusal and buccal surfaces for this study. The resulting acquisitions were treated with MountainsMap® surface imaging and metrology software (Digital Surf). Only surfaces requiring modification to 10% or less of their total surface area were retained for statistical analyses (Schmidt et al., 2019). Surfaces with excessive surface artifacts from the molding process or post‐depositional damage were excluded (Figure 3).

Toothfrax® software (Ungar et al., 2003) was used to calculate complexity (*Asfc*) and anisotropy (*epLsar*). We focus on these two, scale‐sensitive fractal analysis parameters to describe the microwear surfaces, since these variables are frequently considered most useful when discerning differences in diet among various groups (Schmidt et al., 2019; Scott, 2005; Scott et al., 2006).

Complexity, or Area‐scale fractal complexity (*Asfc*), describes surface roughness. For example, enamel surfaces dominated by pits of various sizes and striations overlying each other demonstrate high levels of complexity. This is generally attributed to abrasive diets that include the mastication of hard particles (El‐Zaatari, 2010) or erosion from dietary acids (Hara et al., 2016; Ranjitkar et al., 2017; Krueger et al., 2021). In contrast, lower complexity values indicate simpler surfaces, generally thought to be from softer, more processed, or less grit‐laden diets (Scott et al., 2005). The range of values from human populations tend to be between 1.0 and 2.0 (Mahoney et al., 2016; Schmidt et al., 2015, 2019).

Anisotropy, or length‐scale anisotropy of relief (*epLsar*), documents the alignment of features across the surface and is related to repetitive jaw movements during chewing. High values of anisotropy are often attributed to chewing tough and/or fibrous foods (El‐Zaatari, 2010; Schmidt et al., 2019). In humans, anisotropy values tend to range between 0.0005 and 0.0090 (El‐Zaatari, 2010; Schmidt et al., 2019).

### 3.2 Statistical analysis

All statistical analyses were conducted in R (R Core Team, 2020). A Shapiro–Wilk test for the occlusal and buccal variables indicates a deviation from a normal distribution (Table 1). Ordinary Least Squares (OLS) regressions with 95% confidence intervals are presented along with graphic visualizations of data (ggplot2; Wickham, 2016). Spearman correlations were used to explore the relationship among the different variables and surfaces. Finally, a bootstrap resampling method was applied to compare variables among surfaces. Resampling with replacement was conducted 500 times on the original data. Given the difficulties of obtaining large microwear texture datasets from bioarchaeological contexts—due not only to limitations of absolute sample size, but also the winnowing of the final sample size through the exclusion of individuals with taphonomic surface alterations—the bootstrap method allows probability distributions to be calculated through the resampling of the original data without any assumptions regarding original data distribution. We can draw some statistical conclusions through an improved estimation of sampling distributions that may not be apparent in the data prior to resampling.

**TABLE 1:** Shapiro–Wilk test of distribution for the bootstrap analyses

Surface	Variable	Shapiro–Wilk test bootstrap	Bootstrap *p* value
Occlusal	*Asfc*	0.967	>0.000*
	*epLsar*	0.972	>0.000*
Buccal	*Asfc*	0.948	>0.000*
	*epLsar*	0.969	>0.000*

*Note*: *indicates that the sample is not normally distributed.

Different confocal profilometers can produce different results when examining the same sample (Arman et al., 2016). However, there are no available data for permanent human molars using the confocal profilometer from this study. Furthermore, there is a general lack of open access databases for any human DMTA data. Thus, we used available data (mean and 95% confidence intervals) derived from publications using other confocal profilometers (Karriger et al., 2016; Schmidt et al., 2015; Schmidt et al., 2019; Schmidt et al., 2020; Williams et al., 2020). Table 2 is a general comparative framework for interpreting the diet of the individuals from Cova de la Guineu. Since this is the first study providing buccal surface DMTA data in a bioarchaeological context, no comparative data are available for interpreting our buccal surface DMTA.

**TABLE 2:** Information about the comparative groups. N = number of individuals

Population	N	Site	Chronology	Subsistence	Ref
Foragers	168	Worldwide	—	Global sample of peoples relying primarily on non‐domesticated foods	Schmidt et al., 2019
Farmers	385	Worldwide	—	Global sample of peoples relying on food production	Schmidt et al., 2019
Pastoralist	49	Mongolia	Late Bronze/Iron Age	Herders, Softest diets	Schmidt et al., 2015
Archaic Amerindians	34	Indiana	3500–5500 years BP	Wild and domesticated resource consumption	Schmidt et al., 2020
Early Bronze Age England	21	England	Early Bronze Age	Food production	Schmidt et al., 2015
Bois Madame	12	Belgium	Late Neolithic	Hard food or modest food processing	Williams et al., 2020
Maurenne	18	Belgium	Middle‐Late Neolithic	Hard food or modest food processing	Williams et al., 2020
Sclaigneaux	15	Belgium	Late Neolithic	Hard food or modest food processing	Williams et al., 2020

## 4 RESULTS

Of the 69 LLM2 observed, dental microwear textures were well‐preserved on 33 occlusal surfaces and 39 buccal surfaces, and 27 individuals had well‐preserved microwear textures on both surfaces (e.g., Figure 2). The unavoidable winnowing of our sample due to various taphonomic factors is typical, as noted elsewhere (Correia, et al., 2021; Hernando, Willman, et al., 2020; Krueger, 2016; Martin et al., 2018; Teaford, 2007). The detailed list of occlusal and buccal microwear data by individual can be found in the Supplementary Information (Table S1).

### 4.1 Comparison between surfaces

**FIGURE 4:**
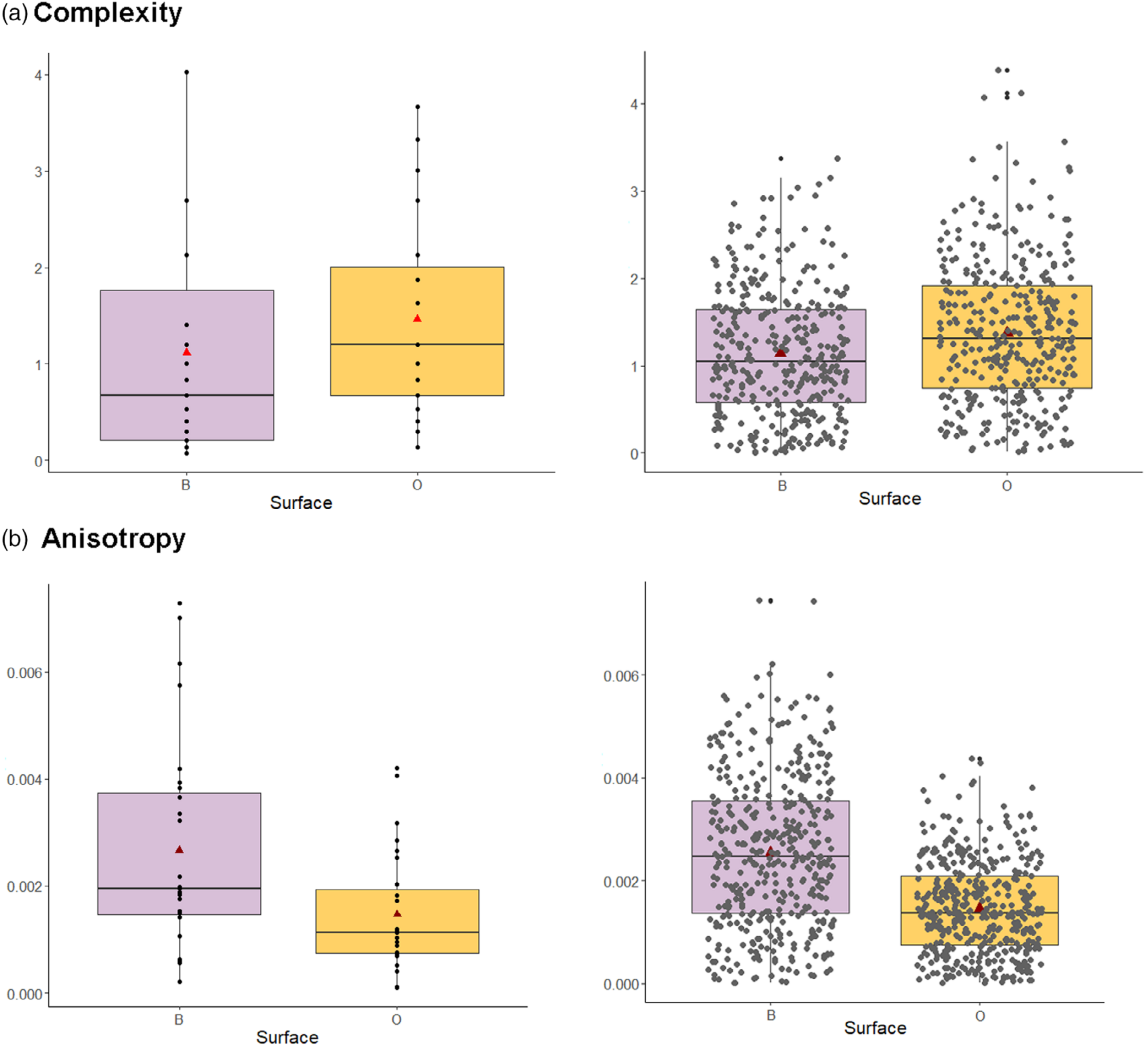
Boxplots of buccal (B) and occlusal (O) surfaces for the variables complexity (*Asfc*) and anisotropy (*epLsar*) from dental microwear texture analysis with their associated bootstrapping analysis (resampling population, n = 500). Red triangles show the means, the horizontal lines represent the medians, each dot is an individual

Descriptive statistics for occlusal and buccal texture microwear are presented in Table 3. Mean complexity is higher for the occlusal surface than for the buccal (Figure 4, Table 3), but mean anisotropy is higher for the buccal surface than the occlusal (Figure 4, Table 3). The application of the bootstrap resampling method shows statistically significant differences between buccal and occlusal complexity as well as buccal and occlusal anisotropy (Figure 4, Table 4).

**TABLE 3:** Summary descriptive statistics for occlusal and buccal texture microwear data from Cova de la Guineu

	Occlusal surface		Buccal surface	
Variable	*Asfc*	*epLsar*	*Asfc*	*epLsar*
Total number	27	27	27	27
Mean	1.47	0.0015	1.12	0.0027
Median	1.20	0.0011	0.92	0.0019
Interquartile range	0.67–2.13	0.0007–0.0020	0.21–2.13	0.0014–0.0038
Standard error	0.19	0.0002	0.21	0.0003
Standard deviation	1.00	0.0011	1.11	0.0019

**TABLE 4:** Bootstrap analyses for test differences between occlusal and buccal surfaces by microwear variable

Variable	Wilcoxon‐Mann–Whitney test value (W)	*p* value
Bootstrap *Asfc*	3128	>0.000*
Bootstrap *epLsar*	1926	>0.000*

*Note*: *indicates that there are significant differences between surfaces (*p <*0.05).

**FIGURE 5:**
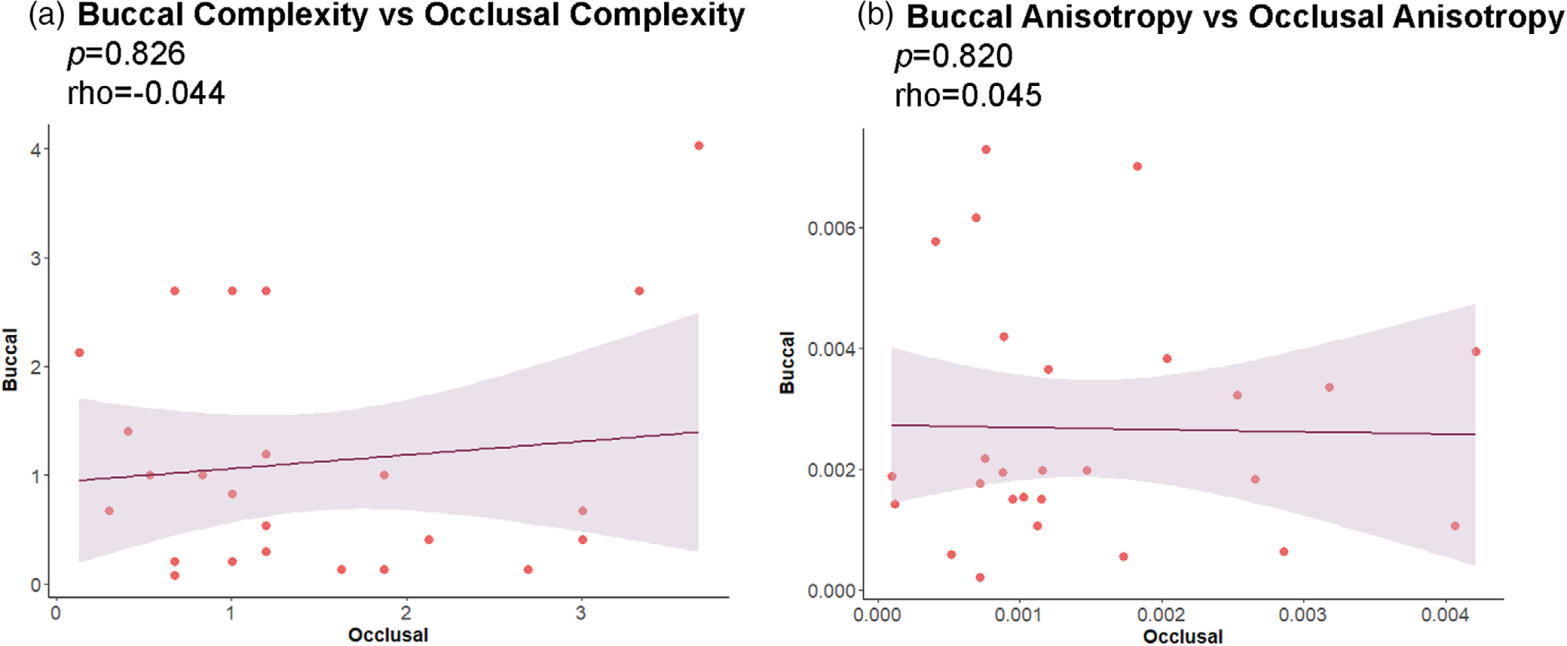
Correlations between buccal and occlusal surfaces for complexity (a) and anisotropy (b). An ordinary Least Square linear regression line is shown for each plot with shaded 95% confidence interval. Spearman correlation (rho) and p‐value are shown for each bivariate plot

**FIGURE 6:**
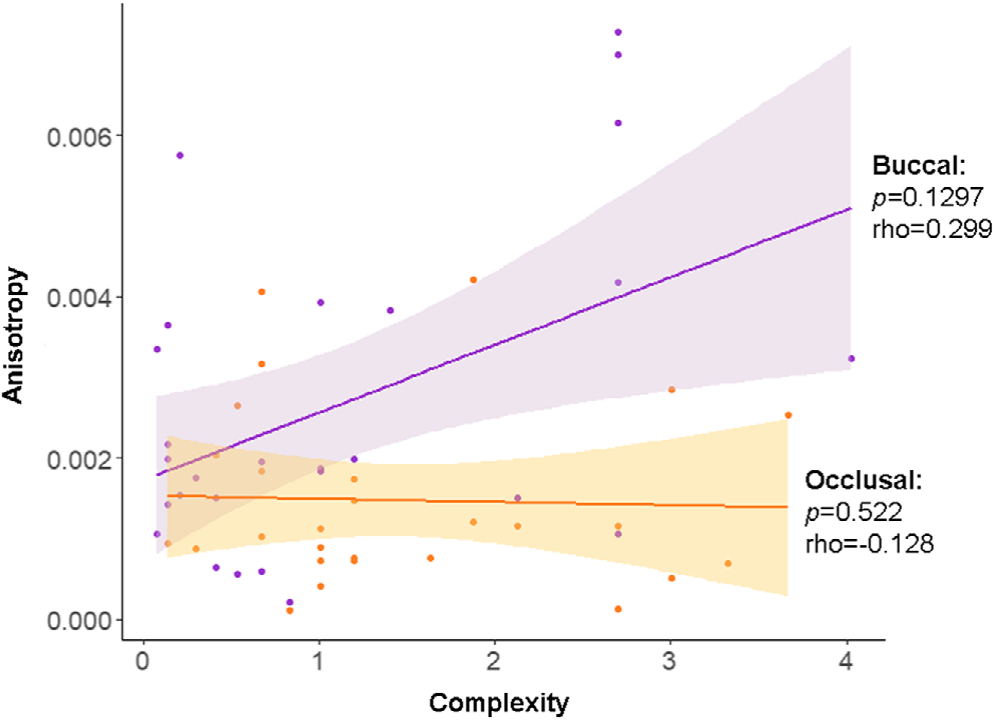
Bivariate plot by individual comparing DMTA variables (*Asfc* and *epLsar*) by surfaces (purple: Buccal; orange: Occlusal). The shaded regions represent the 95% confidence interval. Spearman correlation (rho) and p‐value are shown for each surface

The Ordinary Least Squares regression indicates that there is no correlation at the level of the individual between the surfaces for either of the texture variables (Figure 5). Occlusal surface was set as the independent variable and buccal surface the dependent one. Likewise, there is no correlation between the texture variables for either surface (Figure 6).

### 4.2 Comparison between groups

**FIGURE 7:**
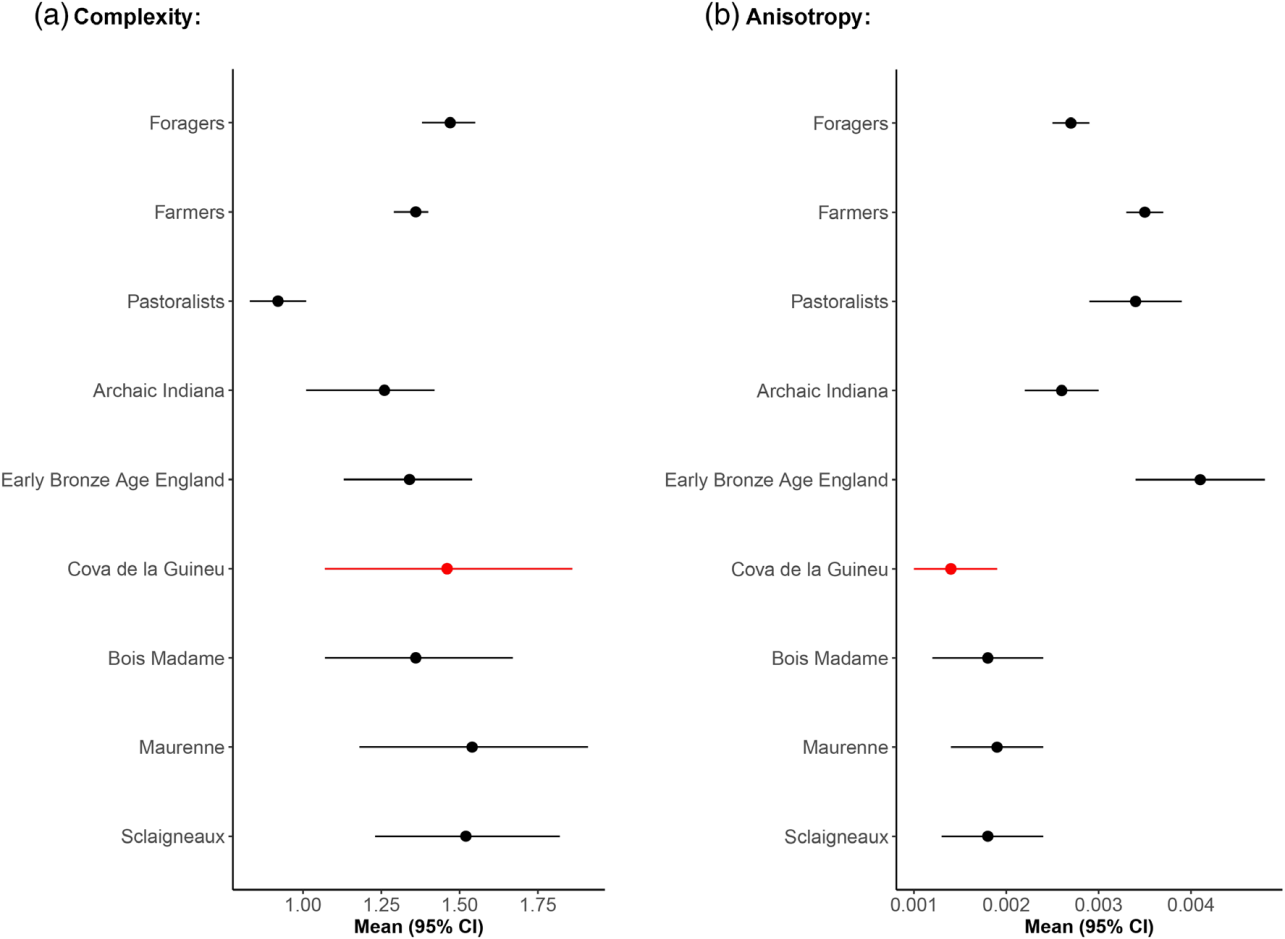
Graphic comparison of means and 95% confidence intervals of complexity (a) and anisotropy (b) on occlusal surfaces for Cova de la Guineu and the comparative samples (see Table 2 for descriptions)

When comparing mean values and confidence intervals with other Holocene groups and macro‐subsistence categories (Figure 7), Cova de la Guineu exhibits a higher mean occlusal complexity value with non‐overlapping 95% CI compared with the Xiongnu pastoralists (Mongolia). Cova de la Guineu has a slightly higher mean complexity than both the forager and farmer macro‐subsistence groups, but the Cova de la Guineu confidence interval completely overlaps with both farmer and forager macrogroups (Figure 7). This is to be expected given the much larger sample sizes in the macro‐subsistence groups compared to that of Cova de la Guineu and the other Holocene comparative groups (Table 2), and the fact that the macro‐subsistence groups are based on global averages that encompass a wide range of chronologies, ecogeographic variation, and culturally specific dietary strategies (Schmidt et al., 2019). Mean occlusal complexity is similar between Cova de la Guineu and the Middle Neolithic populations from Maurenne and Sclaigneaux in Belgium (Table 5).

**TABLE 5:** Descriptive statistic for the comparative samples

	*Asfc*				*epLsar*			
Population	Mean	N	SD	95% CI	Mean	N	SD	95% CI
Foragers	1.47	168	0.58	1.38–1.55	0.0027	181	0.0013	0.0025–0.0029
Farmers	1.36	385	0.57	1.29–1.40	0.0035	392	0.0018	0.0033–0.0037
Pastoralists	0.92	49	0.31	0.83–1.01	0.0034	49	0.0017	0.0029–0.0039
Archaic Indiana	1.26	34	0.47	1.01–1.42	0.0026	34	0.0011	0.0022–0.0030
Early Bronze Age England	1.34	21	0.44	1.13–1.54	0.0041	21	0.0016	0.0034–0.0048
Bois Madame	1.36	12	0.41	1.07–2.42	0.0018	12	0.0009	0.0012–0.0024
Maurenne	1.54	18	0.74	1.18–1.91	0.0019	18	0.0010	0.0014–0.0024
Sclaigneaux	1.52	15	0.53	1.23–1.82	0.0018	15	0.0011	0.0013–0.0024
Cova de la Guineu	1.46	27	1.00	1.07–1.86	0.0014	27	0.0019	0.0010–0.0019

Abbreviations: N, number of individuals analyzed; SD, Standard deviation; 95% CI, confidence interval of mean at 95%.

Cova de la Guineu has the lowest mean occlusal anisotropy values in comparison to others comparative samples but has overlapping 95% CI with each of the Belgian Neolithic samples (Figure 7). While the differences between the macro‐subsistence groups and Cova de la Guineu are to be expected (see reasoning above), the much lower occlusal anisotropy compared to the pastoralists and chronological later food producers from Bronze Age England are notable (see below).

## 5 DISCUSSION AND CONCLUSION

### 5.1 Buccal and occlusal DMTA

Previous occlusal DMTA studies on human populations show that diets high in hard food items and/or large abrasive particles contribute to higher complexities, and softer foods with fewer exogenous hard particle inclusions contribute to lower complexities (e.g., El‐Zaatari, 2010; Mahoney et al., 2016; Schmidt et al., 2015, 2019, 2020). Similarly, Aliaga‐Martínez and colleagues (2017) found that non‐human primates consuming hard and brittle diets have high‐buccal complexity and low‐buccal anisotropy while the opposite is found in non‐human primates eating tough (folivorous) diets. However, since this is the first DMTA study that analyzes the relationship between buccal and occlusal signatures on the same tooth, our interpretations are challenged without a reference framework. For that reason, our complete database was published in an open repository for comparative purposes in the future.

#### 5.1.1 Complexity

Aliaga‐Martínez and colleagues (2017) noted that buccal complexities in non‐human primates were much lower on average than values obtained for the occlusal surfaces (e.g., Scott et al., 2012), which could be largely attributed to an absence of pitting in buccal surfaces (Aliaga‐Martínez et al., 2017). The bootstrap method showed statistically significant differences between buccal and occlusal complexity as well as anisotropy, which could support the assertion of Aliaga‐Martínez and colleagues (2017) that a lack of pitting tends to produce lower complexity values on buccal surfaces. While the utility of buccal complexity remains poorly understood for dietary reconstructions in humans based on our results from one sample, it may be a useful variable for understanding human dietary variation when large, open access, comparative databases are available.

#### 5.1.2 Anisotropy

The significant difference between buccal and occlusal anisotropy values from Cova de la Guineu could be explained by different microwear formation mechanisms on each surface. High‐anisotropy values on the buccal surface could be due to a lack of tooth‐to‐tooth interaction during chewing in contrast to the occlusal surface. This means that abrasive particles, that would create pits on the occlusal surface, behave differently on the buccal surface where they are dragged and pushed across this surface (Lucas et al., 2013). Under this rationale, higher anisotropies on the buccal surfaces relative to the occlusal surfaces could be congruent with the lack of buccal pitting (Hua et al., 2015). Clearly, greater intergroup sampling is needed, to determine the degree to which buccal microwear reflects or complements DMTA signatures on the occlusal surface.

#### 5.1.3 Intra‐facet variation

Note that the measured and analyzed surfaces within the occlusal facet 9 and buccal lower third are small and only account for a small fraction of the whole surface. Such random sampling of dental surfaces that likely display some degree of intra‐surface variation could also influence the assessment of correlations among microwear variables on occlusal and buccal surfaces. Although intra‐surface variation is not usually quantified in dental microwear studies, strong intra‐facet variation was detected on facet 9 of deciduous teeth of *Homo sapiens* juveniles from both archeological and extant populations (Bas et al., 2020). Intra‐facet variation is also well documented in non‐human primates (Krueger et al., 2008) and non‐primate mammals (e.g., Ramdarshan et al., 2017; Schulz et al., 2010). The same sampling bias may apply to buccal surfaces: in traditional SEM methodology, the image acquired at 100× magnification covers a broad patch of enamel (0.56 mm^2^), whereas we used the same area as occlusal DMTA (242 x 182 μm) in this study. Future investigations of correlations among microwear variables measured on occlusal and buccal surfaces should consider the potential effect of intra‐surface variation by measuring several loci per surface (e.g., Ackermans et al., 2021).

#### 5.1.4 Inter‐surface variation

As discussed previously, one of the downsides of 2D and 3D dental microwear analyses, regardless of the type of surface considered, is the large number of samples that must be discarded due to different taphonomic issues, poor molds, and other factors (Correia et al., 2021; Hernando, Willman, et al., 2020; Krueger, 2016; Teaford, 2007). In vivo wear is more pronounced on the occlusal surfaces, making facet 9 more likely to be unusable in analyses. Another complicating factor of buccal surface DMTA is the presence of perikymata (Figure 3D), which affect the quantification of complexity and anisotropy and cannot be removed with the MountainsMap software since they are distributed in the entire studied area. In line with Krueger et al. (2021) concerning occlusal surfaces, we suggest that more experimental studies on the characterization of buccal surface DMTA will improve our understanding of buccal microwear formation, turnover, and interpretation of DMTA variables for dietary reconstruction. Likewise, the combination of paired surface data would be an advantage for refining the diet reconstruction interpretation when both surfaces are available for same tooth. Furthermore, it will also allow the inclusion of more samples since the buccal surface is less affected by in vivo wear (Pérez‐Pérez, 2004, Aliaga‐Martínez et al., 2017), and teeth with high‐occlusal wear could be included in the study by analyzing the buccal surface.

### 5.2 Dietary reconstruction of Cova de la Guineu

As would be expected, the mean occlusal complexity and 95% confidence intervals from Cova de la Guineu were similar to the other food producing groups examined, with the exception of the pastoralist groups. This indicates that Cova de la Guineu diets were significantly harder and/or abrasive than the soft diets of the pastoralist group from Xiongnu (Karriger et al., 2016; Schmidt et al., 2015; Williams et al., 2020), but comparable to the Neolithic groups from Belgium. The relatively high complexity is generally attributed to hard dietary items (Xia et al., 2015) and/or relatively high loads of exogenous grit and abrasives in the diet (Lucas et al., 2013; van Casteren et al., 2020). The addition of anisotropy provides greater dietary discrimination, given that Cova de la Guineu and the Neolithic groups cluster around the lower range of anisotropy compared to the rest of the samples. Low anisotropy is generally attributed to eating hard foods, since hard foods require more forceful, uniform jaw movements to break down compared to the homogeneous jaw movements needed to masticate tough and fibrous foods (Schmidt et al., 2019).

Schmidt et al. (2019) hypothesized that differences in complexity and anisotropy between Early (Neolithic and the Early Bronze Age) and Late (Late Bronze Age to medieval period) food producers would correspond to a technological shift in food processing. While they only found significant differences in terms of complexity between earlier and later food producers, this analysis showed that some of those differences may be perceptible on finer chronological scales from the Neolithic to the Early Bronze Age. For instance, anisotropy for Cova de la Guineu and the Belgian Neolithic groups are clearly differentiated from the Early Bronze Age England sample, which indicates that the latter had softer and more processed diets, containing less large abrasive particles or hard foods.

Previous dietary reconstruction based on δ^13^C and δ^15^N analyses for Cova de la Guineu suggested a mixed diet with the inclusion of C_3_ terrestrial resources and animal intake (Villalba‐Mouco et al., 2018). Here, high‐occlusal complexities suggest high abrasiveness that may be related to the consumption of abundant hard particles such as cereals that contain phytoliths or grit from processing (Gügel et al., 2001; Schmidt et al., 2019; Xia et al., 2015). Similarly, hard and abrasive diets, interpreted as modestly processed, were observed in the contemporaneous Belgian populations (Williams et al., 2020). However, Cova de la Guineu functioned only as a burial cave (Oms et al., 2016), so there are no associated millstones to infer if this abrasive diet is due to coarse processing methods, by the disaggregation of the rock during milling, or hard physical properties of the food consumed, like cereals.

At this point, the buccal surface can provide information corroborating the occlusal results. We cannot rule out the role of food material properties on the formation of the microwear signal (see discussion), but propose that small, hard foods like some cereals could contribute to the occlusal surface pitting. However, the food mechanical properties (e.g., shape) in conjunction with exogenous abrasives (e.g., grit) are also likely contributors to occlusal pitting. Likewise, the same hard food and/or abrasive particles in the bolus would contribute to the high anisotropy on the buccal surface through mastication and compression of the bolus by the tongue and cheek. These movement of the bolus would drag and push hard and/or abrasive particles across the buccal surface and create striations.

Thus, we propose a diet high in exogenous abrasives and/or hard food for the Cova de Guineu individuals based on the combination of occlusal and buccal microwear. This dietary signal is compatible with the mixed diet suggested by isotopic analyses (Villalba‐Mouco et al., 2018) and similar to contemporaneous Belgian populations.

## CONFLICT OF INTEREST

The authors declare no conflict of interest.

### OPEN RESEARCH BADGES

This article has been awarded Open Data Badge for making publicly available the digitally‐shareable data necessary to reproduce the reported results. Data is available at Open Science Framework

### AUTHOR CONTRIBUTIONS

**Raquel Hernando:** Conceptualization (lead); data curation (lead); formal analysis (lead); funding acquisition (equal); investigation (lead); methodology (lead); visualization (lead); writing – original draft (lead). **John C. Willman:** Conceptualization (equal); formal analysis (supporting); funding acquisition (lead); supervision (equal); writing – review and editing (supporting). **Antoine Souron:** Methodology (supporting); software (supporting); writing – review and editing (supporting). **Artur Cebrià:** Funding acquisition (lead); project administration (lead); writing – review and editing (supporting). **F. Xavier Oms:** Funding acquisition (lead); project administration (lead); writing – review and editing (supporting). **Juan I. Morales:** Funding acquisition (lead); project administration (lead); writing – review and editing (supporting). **Marina Lozano:** Formal analysis (supporting); supervision (lead); writing – review and editing (supporting).

## Supporting information


**Figure S1** Four examples of the human remains from Cova de la Guineu, to observe the dental condition.

**Table S1** Detailed list of individual teeth (N = 69) examined in this study. All teeth are lower left second molars (LLM2). DMTA analysis were performed on buccal (B), occlusal (O) or both (BO) enamel surfaces. Poor quality scans with obscured microwear features that were removed from study are indicated by dashes (−) and the reason for removing them is given.

## Data Availability

All data generated in this study is available in the “Supplementary Information files” and in an Open Access Repository (10.5281/zenodo.5213857).
