# Supplementary material for: What about the buccal surfaces? Dental microwear texture analysis of buccal and occlusal surfaces refines paleodietary reconstructions
Source: Am J Biol Anthropol. 2022 Mar 8;178(2):347–59. doi: 10.1002/ajpa.24509 (PMC9313852; doi:10.1002/ajpa.24509)

**Figure S1.** Four examples of the human remains from Cova de la Guineu, to observe the dental condition.
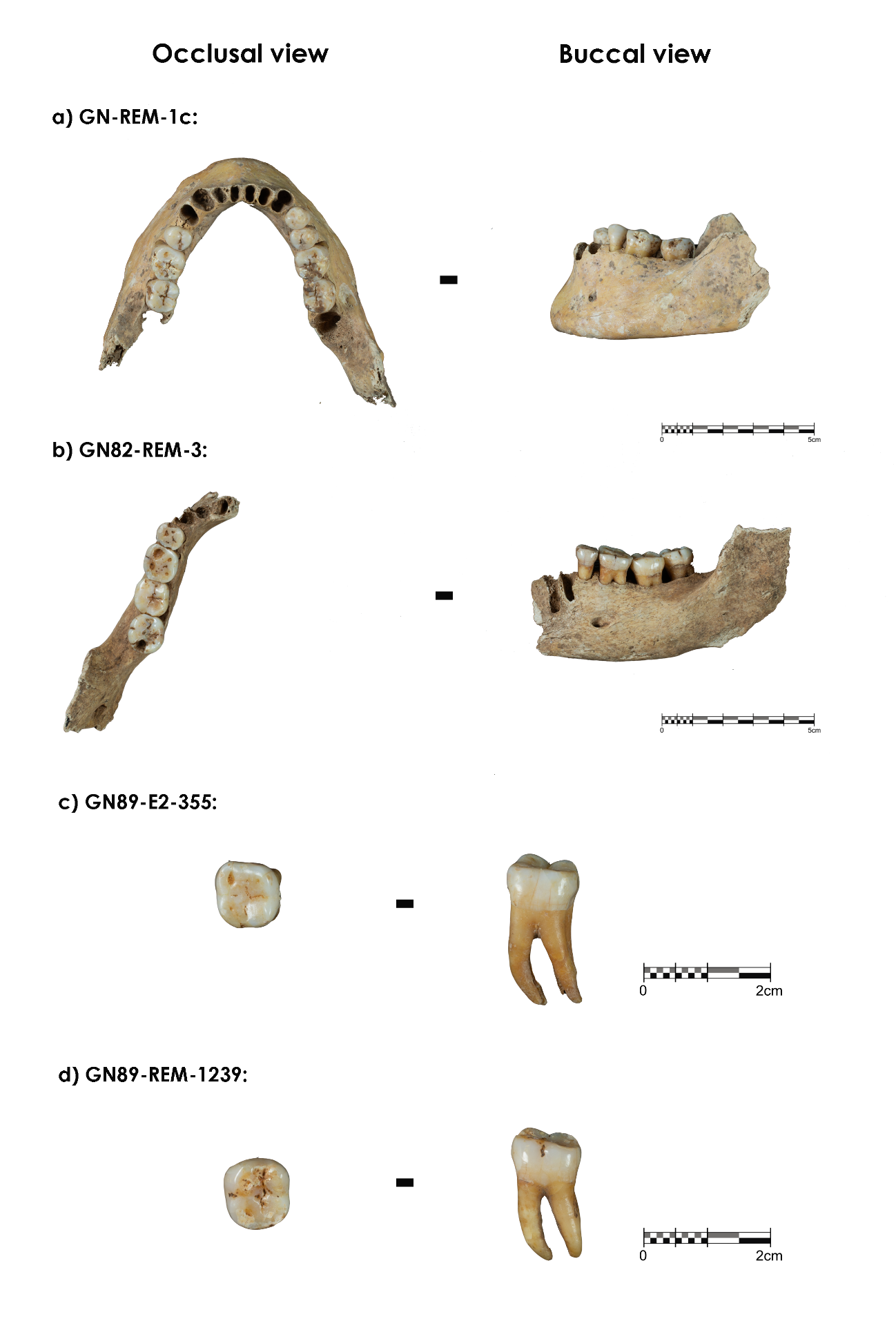

Supplement: Supplementary file 1 — Figure S1 Four examples of the human remains from Cova de la Guineu, to observe the dental condition. [file AJPA-178-347-s002.docx]
