# Supplementary material for: What about the buccal surfaces? Dental microwear texture analysis of buccal and occlusal surfaces refines paleodietary reconstructions
Source: Am J Biol Anthropol. 2022 Mar 8;178(2):347–59. doi: 10.1002/ajpa.24509 (PMC9313852; doi:10.1002/ajpa.24509)
Supplement: Supplementary file 2 — Table S1 Detailed list of individual teeth (N = 69) examined in this study. All teeth are lower left second molars (LLM2). DMTA analysis were performed on buccal (B), occlusal (O) or both (BO) enamel surfaces. Poor quality scans with obscured microwear features that were removed from study are indicated by dashes (−) and the reason for removing them is given. [file AJPA-178-347-s001.docx]

**Table S1.** Detailed list of individual teeth (N = 69) examined in this study. All teeth are lower left second molars (LLM2). DMTA analysis were performed on buccal (B), occlusal (O) or both (BO) enamel surfaces. Poor quality scans with obscured microwear features that were removed from study are indicated by dashes (-) and the reason for removing them is given.

|  |  | **Occlusal** | | | **Buccal** | | |  |
| --- | --- | --- | --- | --- | --- | --- | --- | --- |
| **Specimen Number** | **Surface** | ***Asfc*** | ***epLsar*** | ***Occlusal Removed*** | ***Asfc*** | ***epLsar*** | ***Buccal Removed*** | |
| **GN92-REM-1** | - | - | - | Poor molds | - | - | Postmortem wear | |
| **GN10-REM-L3-2** | - | - | - | Postmortem wear | - | - | Postmortem wear | |
| **GN82-REM-3** | B | - | - | Poor molds | 0.07 | 0.0001 | - | |
| **GN95-REM-5** | - | - | - | Poor molds | - | - | Erosion | |
| **GN-REM-7** | - | - | - | Eruption | - | - | Perikymata | |
| **GN95-REM-9** | B | - | - | Macrowear | 0.13 | 0.0017 | - | |
| **GN89-REM-13** | BO | 0.67 | 0.0032 | - | 0.07 | 0.0033 | - | |
| **GN95-REM-15** | BO | 2.70 | 0.0001 | - | 0.13 | 0.0014 | - | |
| **GN-REM-16** | O | 0.83 | 0.0038 | - | - | - | Broken teeth | |
| **GN01-SUP-G8-17** | - | - | - | Macrowear | - | - | Broken teeth | |
| **GN-REM-19** | O | 3.33 | 0.0010 | - | - | - | Perikymata | |
| **GN-REM-21** | B | - | - | Poor molds | 1.20 | 0.0003 | - | |
| **GN90-F6-IB-24** | BO | 3.00 | 0.0029 | - | 0.41 | 0.0006 | - | |
| **GN95-REM-27** | - | - | - | Eruption | - | - | Perikymata | |
| **GN95-REM-31** | - | - | - | Macrowear | - | - | Postmortem wear | |
| **GN95-REM-32** | - | - | - | Poor molds | - | - | Postmortem wear | |
| **GN95-REM-33** | - | - | - | Macrowear | - | - | Postmortem wear | |
| **GN88-D3-REM-40** | BO | 3.00 | 0.0005 | - | 0.67 | 0.0006 | - | |
| **GN94-REM-43** | B | - | - | Postmortem wear | 1.87 | 0.0007 | - | |
| **GN95-REM-46** | - | - | - | Poor molds | - | - | Postmortem wear | |
| **GN95-REM-48** | B | - | - | Poor molds | 0.41 | 0.0027 | - | |
| **GN95-REM-49** | BO | 1.20 | 0.0007 | - | 0.30 | 0.0018 | - | |
| **GN95-REM-53** | B | - | - | Poor molds | 1.87 | 0.0077 | - | |
| **GN95-REM-55** | BO | 0.67 | 0.0018 | - | 2.70 | 0.0070 | - | |
| **GN95-REM-58** | - | - | - | Macrowear | - | - | Postmortem wear | |
| **GN82-81** | BO | 2.13 | 0.0012 | - | 0.41 | 0.0015 | - | |
| **GN08-REM-97** | - | - | - | Eruption | - | - | Perikymata | |
| **GN-B6-113** | BO | 1.20 | 0.0008 | - | 2.70 | 0.0073 | - | |
| **GN-E1-132** | BO | 0.53 | 0.0027 | - | 1.01 | 0.0018 | - | |
| **GN-D4-140** | BO | 2.70 | 0.0012 | - | 0.13 | 0.0020 | - | |
| **GN95-B6-161** | - | - | - | Poor molds | - | - | Postmortem wear | |
| **GN88-D3-201** | - | - | - | Macrowear | - | - | Postmortem wear | |
| **GN89-D1-343** | B | - | - | Poor molds | 0.41 | 0.0009 | - | |
| **GN89-E2-355** | BO | 1.01 | 0.0004 | - | 0.21 | 0.0058 | - | |
| **GN09-REM-M3-429** | - | - | - | Eruption | - | - | Perikymata | |
| **GN88-E3-431** | - | - | - | Poor molds | - | - | Postmortem wear | |
| **GN89-D1-489** | - | - | - | Poor molds | - | - | Postmortem wear | |
| **GN97-B3-555** | BO | 1.63 | 0.0008 | - | 0.13 | 0.0022 | - | |
| **GN97-B3-556** | BO | 0.41 | 0.0020 | - | 1.41 | 0.0038 | - | |
| **GN89-REM-563** | - | - | - | Taphonomic and erosion | - | - | Taphonomic and erosion | |
| **GN89-D1-668** | BO | 1.20 | 0.0017 | - | 0.53 | 0.0006 | - | |
| **GN-REM-687** | - | - | - | Taphonomic and erosion | - | - | Postmortem wear | |
| **GN89-REM-688** | BO | 1.87 | 0.0042 | - | 1.01 | 0.0039 | - | |
| **GN89-REM-700** | - | - | - | Macrowear | - | - | Broken teeth | |
| **GN96-B6-955** | BO | 0.83 | 0.0001 | - | 1.01 | 0.0019 | - | |
| **GN95-C7-1039** | - | - | - | Macrowear | - | - | Postmortem wear | |
| **GN89-REM-1239** | B | - | - | Taphonomic and erosion | 1.20 | 0.0050 | - | |
| **GN89-REM-1555** | - | - | - | Macrowear | - | - | Broken teeth | |
| **GN90-D1-1564** | BO | 0.67 | 0.0041 | - | 0.07 | 0.0011 | - | |
| **GN13-REM-O5-1674** | BO | 1.01 | 0.0011 | - | 2.70 | 0.0011 | - | |
| **GN91-REM-3301** | BO | 3.33 | 0.0007 | - | 2.70 | 0.0062 | - | |
| **GN89-REM-3496** | BO | 0.30 | 0.0009 | - | 0.67 | 0.0020 | - | |
| **GN-REM-3501** | - | - | - | Poor molds | - | - | Postmortem wear | |
| **GN-REM-3580** | B | - | - | Poor molds | 0.67 | 0.0030 | - | |
| **GN89-REM-3592** | BO | 1.20 | 0.0015 | - | 1.20 | 0.0020 | - | |
| **GN89-REM-3610** | BO | 3.67 | 0.0025 | - | 4.03 | 0.0032 | - | |
| **GN89-E2-3796** | - | - | - | Macrowear | - | - | Postmortem wear | |
| **GN95-REM-1A** | BO | 1.87 | 0.0012 | - | 0.13 | 0.0037 | - | |
| **GN91-REM-3303** | B | - | - | Poor molds | 0.41 | 0.0065 | - | |
| **GN89-REM-3611** | - | - | - | Erosion | - | - | Postmortem wear | |
| **GN89-REM-3612E** | O | 0.83 | 0.0004 | - | - | - | Perikymata | |
| **GN-REM-39** | - | - | - | Erosion | - | - | Erosion | |
| **GN-REM-mandible-3** | B | - | - | Poor molds | 0.13 | 0.0019 | - | |
| **GN88-RE-D3-40-1** | O | 3.00 | 0.0005 | - |  |  | Perikymata | |
| **GN-M59-59D** | BO | 0.13 | 0.0009 | - | 2.13 | 0.0015 | - | |
| **GN89-REM-686B** | BO | 1.01 | 0.0007 | - | 0.83 | 0.0002 | - | |
| **GN-REM-1C** | BO | 1.01 | 0.0009 | - | 2.70 | 0.0042 | - | |
| **GN-REM-10** | - | - | - | Poor molds | - | - | Postmortem wear | |
| **GN-REM-22** | BO | 0.67 | 0.0010 | - | 0.21 | 0.0015 | - | |
